# Reliability of and Relationship between Flight Time to Contraction Time Ratio and Reactive Strength Index Modified

**DOI:** 10.3390/sports6030081

**Published:** 2018-08-15

**Authors:** John J. McMahon, Jason P. Lake, Paul Comfort

**Affiliations:** 1 Directorate of Sport, Exercise and Physiotherapy, University of Salford, Frederick Road, M6 6PU Salford, UK; p.comfort@salford.ac.uk; 2 Department of Sport and Exercise Sciences, University of Chichester, PO19 6PE Chichester, UK; j.lake@chi.ac.uk

**Keywords:** countermovement jump, jump strategy, jump height, force platform, jump monitoring

## Abstract

Countermovement jump (CMJ) force-time testing is commonly used to monitor seasonal alterations in athletes’ CMJ strategy (to infer alterations in neuromuscular function). The flight time to contraction time (FT:CT) ratio and reactive strength index modified (RSI_mod_) are considered to be two primary CMJ variables of interest. Due to similar calculations, it is likely that the FT:CT ratio and RSI_mod_ share similar reliability and an almost perfect relationship. Consequently, there may be no requirement to include both variables in CMJ monitoring reports. This study aimed to investigate this by recruiting twenty-five males to perform three CMJs on a force platform across two sessions that were separated by one week. The FT:CT ratio and two calculations of RSI_mod_ (based on the jump height from either flight time or take-off velocity) were then calculated using robust methods. The between-day reliability was good-excellent for all of the variables (95% confidence interval range of the coefficient of variation = 2.02–9.22%) with no significant between-day differences noted (*p* ≥ 0.231). There was an almost perfect positive relationship between the FT:CT ratio and RSI_mod_ regardless of the calculation method (*r* = 0.944–0.947, *p* < 0.001). As the FT:CT ratio and RSI_mod_ yield similar absolute reliability and share 90% of common variance, there is little reason to include both variables in CMJ monitoring reports.

## 1. Introduction 

Countermovement jump (CMJ) testing via force platform analysis is now commonplace across a variety of sports settings in addition to many sports science research studies [1]. The rationale for this type of assessment is that changes in jump strategy (i.e., the underpinning force and time characteristics) that lead to either changes in or maintenance of jump height (JH) are thought to provide insight into alterations in neuromuscular function, namely due to supercompensation or fatigue [2,3]. One simple method of describing the CMJ strategy is to report the time between the onset of the movement (i.e., the start of the unweighting phase [1]) and take-off (sometimes referred to as time to take-off [TTT] [4] or contraction time [CT] [5]) and JH, or alternative measures of JH like flight time (FT, time spent in the CMJ flight phase). Further, calculating either the ratio between FT and CT or the ratio between JH and TTT yields the FT:CT ratio and the reactive strength index modified (RSI_mod_), respectively [4,5]. The FT:CT ratio has mainly been utilized as an indirect measure of monitoring neuromuscular fatigue in team sport athletes [6,7,8]. The RSI_mod_ has mainly been used to compare neuromuscular function between different athletic groups [9,10,11,12], although it has recently been suggested that it might also be a useful method of indirectly monitoring neuromuscular fatigue due to its proposed similarities to the FT:CT ratio [13]. The relationship between the FT:CT ratio and RSI_mod_ is, however, currently unknown. 

As mentioned above, both the FT:CT ratio and RSI_mod_ include CT (hereafter used to describe TTT too) in their respective calculations. Any errors in the way CT is identified could, therefore, negatively affect both the FT:CT ratio and RSI_mod_ calculations. The CT could be influenced by the force thresholds that are used to determine the onset of movement and take-off [14,15], as well as the variability from the athletes’ movement strategy [16]. Similarly, because the FT:CT ratio uses FT in its calculation, it may be influenced by the force thresholds that are used to identify take-off and touchdown and any variation between the take-off and touchdown leg joint geometry (i.e., it will be prolonged if subjects flex their ankles, knees, or hips during the flight phase [17]). Any errors in the quantification of FT will then be exacerbated if it is used to estimate JH, because a key part of this involves squaring FT [18]. Thus, if using the FT method to estimate JH to then calculate RSI_mod_, one can expect the error to be larger for RSI_mod_ compared with the FT:CT ratio. Jump height can also be estimated from vertical take-off velocity (TOV), however this may be affected by the force threshold that is used to identify take-off and how body weight (and then body mass) is measured [14], as the force-time record must be numerically integrated to either yield net propulsion impulse (and then divide this by body mass) or centre of mass velocity across the entire jump. Any errors in TOV will also be amplified when it is used to estimate JH because a key part of this involves squaring TOV [18]. If robust CMJ force-time data collection procedures are adhered to, however, the error that is associated with TOV should be less than those that are associated with FT. Researchers have also demonstrated that the force platform sample frequency can influence most of the above-mentioned factors, such as force thresholds, bodyweight measurement, and JH, with 1000 Hz suggested to be preferable [14,15]. The above factors could influence the validity, and possibly the reliability, of the FT:CT ratio and RSI_mod_ calculations and so this must be considered when using them in both research studies and applied practice.

To the authors’ knowledge, only four studies have assessed the between-day reliability of the FT:CT ratio [3,5,19,20], while only one study has determined the between-day reliability of RSI_mod_ [19]. Of the four studies to include between-day reliability statistics for the FT:CT ratio, three of them used an onset of movement threshold that was equal to 5% of bodyweight [3,5,19], however only one of these explained how they determined bodyweight [19] and they used the suggested criterion method to identify the start of the unweighting phase [15]. Two of the studies provided no information on the take-off and touchdown force thresholds [3,20], whereas one used arbitrary values of 5 N for take-off and 50 N for touchdown [5]. Kennedy and Drake [19] used a superior method of identifying take-off and touchdown, using the peak residual force in the flight phase as the threshold to determine both events. The JH calculation was not disclosed in two of these studies [5,15], whereas it was calculated from peak velocity (it is unclear whether this was derived from the numerical integration of the force-time record or from a linear position transducer) by Gathercole et al. [3] which would have overestimated JH due to the peak velocity occurring before take-off [1]. Kennedy and Drake [19] used the preferred method of calculating JH from vertical TOV [21]. Additionally, only Kennedy and Drake [19] used the suggested minimum force platform sample frequency of 1000 Hz [14,15], whereas 600 Hz [20] and 200 Hz [3,5] were used in the other studies. Sampling below 1000 Hz can lead to an underestimation of JH [14,15]. 

Despite the methodological shortcomings (particularly in relation to validity) of some of the studies discussed above, acceptable between-day reliability (coefficient of variation [CV] ≤ 5.8%) was reported for the FT:CT ratio by Roe et al. [20] and Gathercole et al. [3], however it was not by Cormack et al. [5] who reported a between-day CV for the FT:CT ratio of 10.3%. The between-day CV% may have been higher in the study by Cormack et al. [5] because only one CMJ trial was performed during each testing occasion. Roe et al. [20] showed that the between-day reliability of the FT:CT ratio only achieved acceptability when the best of 2 or 3 CMJ trials that were performed during each testing occasion were considered. More recently, Kennedy and Drake [19] reported that taking the average rather than the best JH that was obtained across 2–3 CMJ trials further improved its between-day reliability, although they did not report exact CV% values, but rather they presented the CV% in figures. Upon visually inspecting these figures, RSI_mod_ demonstrated marginally worse between-day reliability than the FT:CT ratio (~6.0% vs. ~5.5%), but a slightly better signal-to-noise ratio (derived by dividing the smallest worthwhile change [signal] by the CV [noise]) [19]. Of the constituent variables of the FT:CT ratio and RSI_mod_, FT demonstrated the lowest CV (1.1–3.3%) across the studies [3,5,20], followed closely by JH (CV ≤ 4.6–5.0%). Only Gathercole et al. [3] reported the between-day reliability of CT, showing that it was slightly worse (CV = 6.1%) than the FT:CT ratio (CV = 5.2%). 

Although Kennedy and Drake [19] included both the FT:CT ratio and RSI_mod_ in their study, they only compared the reliability of each measure rather than exploring the relationship between them. Exploring both the reliability of the FT:CT ratio and RSI_mod_ (and their constituent parts) when obtained by using robust methods of CMJ force-time analyses and then studying the relationships between the FT:CT ratio and RSI_mod_ and their constituent parts, formed the purpose of this study. Understanding precisely how much common variance is shared between the FT:CT ratio and RSI_mod_ will help to explain whether these variables describe the same CMJ characteristics (and, thus, whether just one rather than both of the variables should be considered in future work). Comparing between-day reliability statistics will help to identify the variable that is the most sensitive at detecting changes in neuromuscular function. A secondary purpose of this study was to determine the reliability of and the relationship between RSI_mod_ when calculated from JH that is derived from FT (RSI_mod_^FT^) and TOV (RSI_mod_^TOV^). It was hypothesized that (1) FT would be the most reliable variable, thus the FT:CT ratio would yield marginally better reliability than RSI_mod_ (for both methods), (2) the FT:CT ratio and RSI_mod_ (both methods) would share an almost perfect positive relationship and (3) RSI_mod_^TOV^ would yield marginally better reliability than RSI_mod_^FT^ (due to the FT method of calculating JH showing greater error than the TOV method), however they too would share an almost perfect positive relationship. 

## 2. Materials and Methods

### 2.1. Subjects

This study employed a repeated measures and correlational design, whereby twenty-five male sports science students (age = 19.9 ± 2.7 years, height = 1.74 ± 0.05 m, and body mass = 73.7 ± 12.2 kg) performed three CMJs on a force platform on two separate occasions (held at the same time of the day and separated by one week). This enabled between-day reliability of and the relationship between the FT:CT ratio and RSI_mod_ (and their constituent parts) to be determined. All of the subjects competed in team sports at an amateur level (including at least one sports-specific training session and one competitive match per week), performed unsupervised resistance training at least twice weekly (and had done so for at least one year), and had previous experience of performing CMJs in line with the protocols that were discussed in the procedures section. Written informed consent was provided before testing, and the study was pre-approved by the institutional review board (reference number: HST1516-233) and conformed to the World Medical Association’s Declaration of Helsinki.

### 2.2. Procedures

Following a brief (approximately 10 min) warm-up consisting of dynamic stretching and sub-maximal jumping (five sets of single effort and two sets of five repeated CMJs), the subjects performed three recorded CMJs to a self-selected depth [19]. All of the sub-maximal jumps that were completed in the warm-up were also executed to the subjects’ self-selected depth (thus, the repeated CMJs were not performed with the intention of minimizing ground contact time), however they were not performed to a maximal JH. The recorded CMJs were performed approximately three minutes after the completion of the warm-up and each of the three trials were separated by one minute of rest. The subjects were instructed to perform the tested jumps as fast and as high as possible, whilst keeping their arms akimbo. Any jumps that were inadvertently performed with the inclusion of arm swing or leg tucking during the flight phase (tester observation) were omitted and additional jumps were performed after one minute of rest.

### 2.3. Data Collection

Ground reaction force (GRF) was recorded at 1000 Hz using a Kistler (type 9286AA, Kistler Instruments Inc., Amherst, NY, USA) force platform and Bioware 5.11 software (Kistler Instruments Inc., Amherst, NY, USA). The subjects were instructed to stand still for the initial one second of data collection [15,21] to enable the subsequent determination of their body weight (vertical GRF averaged over the first 1 s). The raw vertical force-time data were exported as text files and was analyzed using a customized Microsoft Excel spreadsheet (version 2016, Microsoft Corp., Redmond, WA, USA). 

### 2.4. Data Analysis

The vertical center of mass velocity was determined by dividing vertical GRF (minus body weight) by body mass and then integrating the product using the trapezoid rule [21]. Onset of movement was identified in line with current recommendations [15]. In brief, the standard deviation of the vertical GRF during the first 1 s of data collection was multiplied by five to create a force threshold and the onset of movement was considered to have occurred 30 ms before the initial vertical GRF, representing that body weight, was reduced by this amount. Take-off and touchdown were identified when the vertical GRF fell below and exceeded five times the standard deviation of the flight phase force, respectively [12,21,22]. The flight phase force was identified as the force during the middle of the flight phase (i.e., when the force platform was unloaded) in line with the method that was described by Lake et al. [23,24]. The CT was calculated as the time interval between the onset of movement and take-off. The FT was calculated as the time interval between take-off and touchdown. The FT:CT ratio was calculated as FT divided by CT [7]. Two calculations of JH were performed, one based on FT (JH^FT^) and one based on vertical TOV (JH^TOV^) [21]. For each method (i.e., RSI_mod_^FT^ and RSI_mod_^TOV^), the RSI_mod_ was calculated as the respective JH divided by CT [4].

### 2.5. Statistical Analysis

A two-way mixed-effects model (average measures) intraclass correlation coefficient (ICC), along with the upper and lower 95% confidence interval (CI), was used to determine the relative between-day reliability of each variable. Based on the 95% CI of the ICC estimate, values less than 0.5, between 0.5 and 0.75, between 0.75 and 0.90, and greater than 0.90 were indicative of poor, moderate, good, and excellent relative reliability, respectively [25]. The absolute between-day reliability of each variable was calculated using the CV% (calculated in this study as the standard deviation divided by the mean which was then expressed as a percentage), along with the upper and lower 95% CI. A CV of ≤10% and ≤5% has been used as an indicator of reliability in previous similar studies [5,20]. Due to a lack of consistency across the studies, ≤5% and 5–10% thresholds (based on the 95% CI of the CV% estimate) were considered to represent good and excellent reliability, respectively, in the present study. All of the variables met parametric assumptions, thus the mean between-day differences in all of the variables, along with the mean differences in both JH^TOV^ and JH^FT^ and RSI_mod_^FT^ and RSI_mod_^TOV^, were compared using the dependent t-test. Effect size (ES) calculations (Cohen’s *d*) provided a measure of the magnitude of the differences in each variable (between days and between methods) and they were interpreted as trivial (<0.19), small (0.20–0.49), moderate (0.50–0.79), or large (>0.80). Relationships between the FT:CT ratio and RSI_mod_ (both methods), JH that was estimated from FT and TOV, and RSI_mod_^FT^ and RSI_mod_^TOV^ were explored using the Pearson correlation coefficient. Correlation coefficients were interpreted as trivial (0–0.09), small (0.10–0.29), moderate (0.30–0.49), large (0.50–0.69), very large (0.70–0.89), and almost perfect (0.90–1) [26]. All of the statistical tests were performed using SPSS software (version 23; SPSS Inc., Chicago, IL, USA) with the alpha level set at *p* ≤ 0.05.

## 3. Results

**Figure 1:**
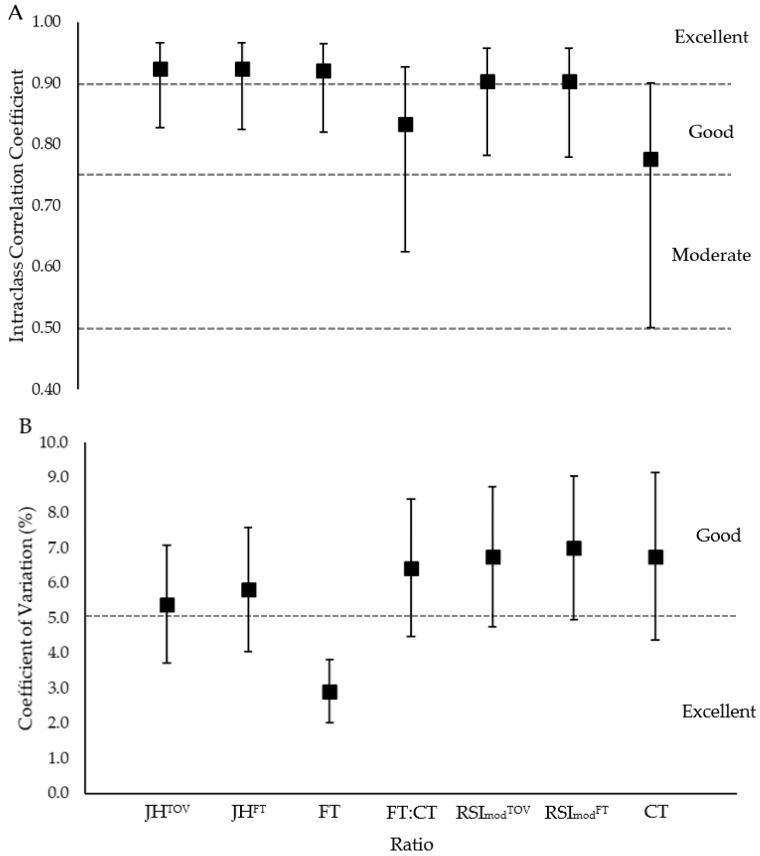
Between-day relative (**A**) and the absolute (**B**) reliability of all of the variables (where: JH = jump height, TOV = take-off velocity, FT = flight time, CT = contraction time, RSI_mod_ = reactive strength index modified). The error bars represent the upper and lower 95% confidence intervals. Grey dashed lines represent the different thresholds (see the statistical analysis section) that were used to interpret the magnitude of the observed reliability values.

Based on the ICC results, the between-day relative reliability was good-excellent for JH^TOV^, JH^FT^, FT, RSI_mod_^FT^, and RSI_mod_^TOV^, however it was moderate-excellent for the FT:CT ratio and CT (Figure 1A). Based on the CV% results, the between-day absolute reliability was excellent for FT, however it was good-excellent for all of the other variables, except for RSI_mod_^FT^ which was good (Figure 1B). There were no significant between-day differences between the means of any variable (*p* ≥ 0.231), with trivial ES noted (≤0.15) for all of the variables except for CT which showed a small between day effect (*d* = 0.21). 

**Figure 2:**
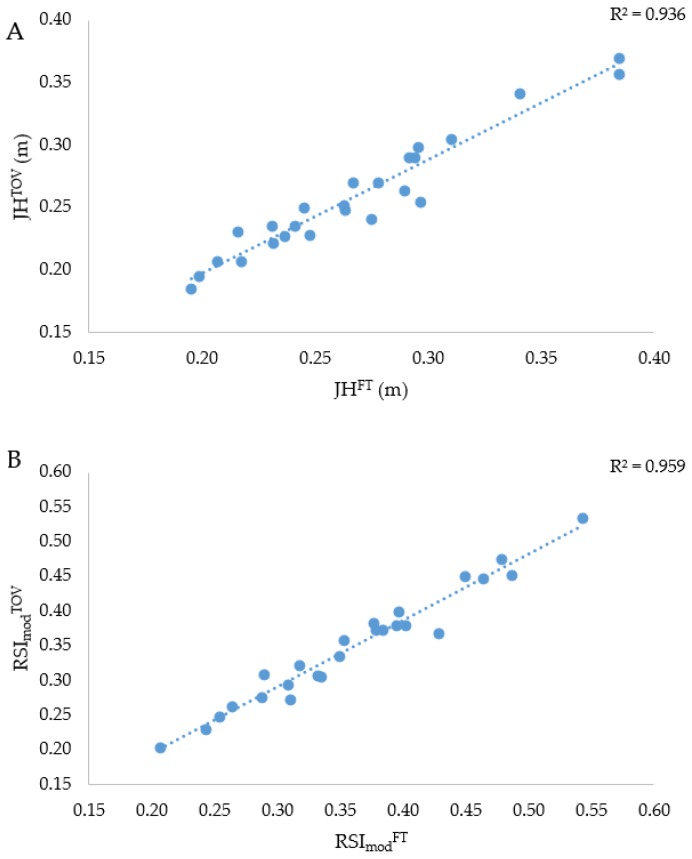
Relationships between JH^TOV^ and JH^FT^ (**A**) and RSI_mod_^TOV^ and RSI_mod_^FT^ (**B**) (where: JH = jump height, TOV = take-off velocity, FT = flight time, CT = contraction time, RSI_mod_ = reactive strength index modified).

There was a significant (*p* = 0.002) but trivial (*d* = 0.18) difference between JH^TOV^ and JH^FT^ (0.26 ± 0.05 m vs. 0.27 ± 0.05 m) of 4.30% (95% CI = 2.78–5.83%), however they shared a significant and almost perfect positive relationship (*r* = 0.969, *p* < 0.001), as shown in Figure 2A. There was also a significant (*p* = 0.002) but trivial (*d* = 0.14) difference between RSI_mod_^TOV^ and RSI_mod_^FT^ (0.35 ± 0.08 vs. 0.36 ± 0.08) of 4.32% (95% CI = 2.80–5.84%), and they too shared a significant and almost perfect positive relationship (*r* = 0.980, *p* < 0.001), as shown in Figure 2B. 

**Figure 3:**
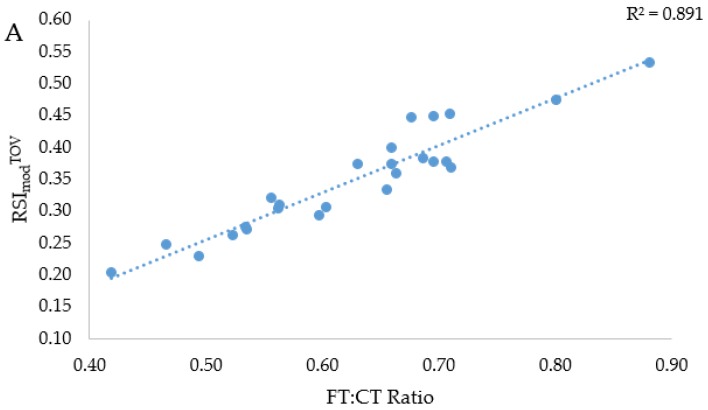

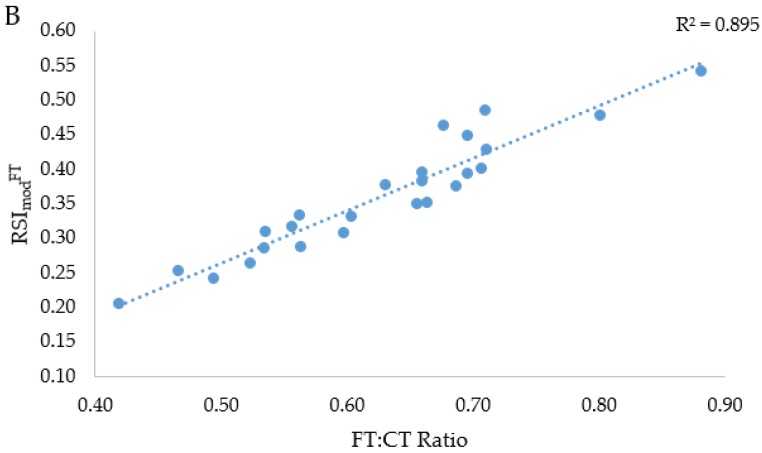
Relationships between RSI_mod_^TOV^ and the FT:CT ratio (**A**) and RSI_mod_^FT^ and the FT:CT ratio (**B**) where: RSI_mod_ = reactive strength index modified, TOV = take-off velocity, FT = flight time, CT = contraction time).

There was a significant and almost perfect positive relationship between RSI_mod_^TOV^ and the FT:CT ratio (*r* = 0.944, *p* < 0.001) and between RSI_mod_^FT^ and the FT:CT ratio (*r* = 0.947, *p* < 0.001), as shown in Figure 3A,B.

## 4. Discussion

The primary aim of this study was to explore both the reliability of and the relationship between the FT:CT ratio and RSI_mod_ (and their constituent parts). The first hypothesis that was related to the primary aim was that FT would be the most reliable variable, thus that the FT:CT ratio would yield marginally better reliability than RSI_mod_ (for both methods). The lower 95% CI of the ICC was lowest for CT (0.501) and was the second lowest for the FT:CT ratio (0.625), with each of these lower bounds being the only ones to fall within the ‘moderate reliability’ range (Figure 1A). These ICC results indicate that the relative (i.e., rank order) between-day reliability was poorest for CT and was the second poorest for the FT:CT ratio, whereas all of the other variables were classed as achieving good-excellent reliability (Figure 1a). When monitoring individual and group changes in CMJ performance between the testing sessions, however, as is often done with athletes, it is the absolute between-day reliability (i.e., CV%) that is of greater interest. The upper 95% CI of the CV% was highest for CT (9.22) and was the second highest for RSI_mod_^FT^ (8.99), whereas the lower 95% CI of the CV% was lowest for FT (2.02) and was the second lowest for JH^TOV^ (3.71) (Figure 1B). Interestingly, despite having the greatest upper 95% CI, the lower 95% CI for CT CV% ranked in the middle (fourth out of the seven reported variables). This highlights a greater range of between-subject variability in CT, compared to the other variables, for the present cohort. Due to FT being the most reliable variable, as was reported in previous work [3,5,20], the FT:CT ratio demonstrated marginally better CV% values than each of the RSI_mod_ calculations (Figure 1B), thus the first hypothesis of the study was accepted. The latter results are in line with a recent study by Kennedy and Drake [19] who used similar methods to the present study and also reported slightly better between-day reliability for the FT:CT ratio compared with RSI_mod_^TOV^ (CV% = ~5.5 vs. ~6.0). The CV% for the FT:CT ratio reported here and by Kennedy and Drake [19] is similar to the values that were reported by Roe et al. [20] and Gathercole et al. [3], however it is substantially better than the value of 10.3% that was reported by Cormack et al. [5]. The poorer absolute reliability that was reported in the latter study was likely due to the unmatched and arbitrary force values that were used to detect take-off and touchdown, in addition to including just one CMJ trial during each testing occasion [5]. 

The second hypothesis that related to the primary aim was that the FT:CT ratio and RSI_mod_ (both methods) would share an almost perfect positive relationship. As hypothesized, an almost perfect positive relationship was seen between the FT:CT ratio and RSI_mod_ despite the method of calculation (Figure 3A,B). The RSI_mod_^FT^ explained 0.4% more of the variance in the FT:CT ratio than the RSI_mod_^TOV^ (Figure 3A,B). The very marginally greater shared variance between RSI_mod_^FT^ and the FT:CT ratio is likely due to FT featuring in both calculations. Nevertheless, 89–90% of the variance in the FT:CT ratio could be explained by RSI_mod_^TOV^ and RSI_mod_^FT^, meaning that the FT:CT ratio and RSI_mod_ essentially describe the same CMJ characteristics, irrespective of the calculation that was used to derive RSI_mod_. In other words, if the FT:CT ratio was to increase between testing occasions, then it is very likely that the RSI_mod_ would have increased also. This is the first study to report the relationship between the FT:CT ratio and RSI_mod_, the purpose of which was to determine whether just one rather than both of the variables should be reported in future studies and applied practice. From a practical standpoint, these results suggest that if the CMJ test is conducted in line with the present study, either the FT:CT ratio or RSI_mod_ can be used to compare athletes or to longitudinally monitor changes in an athlete’s CMJ force-time characteristics, and so there is little point in reporting changes in both variables in studies or to the athletes’ key training team (if being used in applied practice). The only way in which an increase in the FT:CT ratio would markedly outweigh an increase in RSI_mod_^TOV^ would be if an athlete prolonged the FT by excessively flexing their lower limb joints during the flight phase. This highlights the importance of coaching athletes prior to CMJ testing to ensure that they minimize ankle, knee, and hip flexion during the flight phase. In such instances of athletes noticeably flexing ankles, knees, and hips prior to touchdown, it is suggested that the RSI_mod_^TOV^ should be preferentially reported [17]. Additionally, it can be incredibly useful to report the constituent parts of either the FT:CT ratio or RSI_mod_ (depending on which of these variables is reported), along with the mean force, to explain how any changes in their values between the testing occasions have occurred. 

The secondary purpose of this study was to determine the reliability of and the relationship between RSI_mod_ when calculated from JH derived from FT (RSI_mod_^FT^) and TOV (RSI_mod_^TOV^). It was hypothesized that RSI_mod_^TOV^ would yield marginally better reliability than RSI_mod_^FT^ but that they would share an almost perfect positive relationship. From a reliability standpoint, the TOV-derived calculations demonstrated slightly greater reliability (Figure 1A,B), as hypothesized. The JH^FT^ was ~1 cm higher than the JH^TOV^, which equated to a trivial 4.30% difference and is similar to the difference between the calculations that have been reported in previous work [21]. The difference between JH^FT^ and JH^TOV^ resulted in an almost identical trivial difference between RSI_mod_^TOV^ and RSI_mod_^FT^ of 4.32%, with the former yielding a marginally lower score. As hypothesized, however, almost perfect positive relationships were observed between both JH^TOV^ and JH^FT^ and between RSI_mod_^FT^ and RSI_mod_^TOV^, which equated to around 94–96% shared variance (Figure 2A,B). However, it should be remembered that lower-body movement strategies were watched stringently throughout the flight phase and that a failure to do this could yield dramatically different results [27,28]. Despite the differences and the relationships between FT- and TOV-derived calculations being trivial and almost perfect, respectively, it would be prudent to only compare longitudinal changes in athletes’ JH and RSI_mod_ scores if the same calculation is applied during each testing occasion (i.e., using either TOV or FT methods consistently and not using them interchangeably). As a marginally better reliability was attained when using TOV to estimate JH and then RSI_mod_, it perhaps should be the variable of choice in future work. Aside from reliability reasons, and as was alluded to in the previous paragraph, the TOV-derived calculations are unaffected by athletes prolonging their FT by flexing their lower limb joints during the flight phase [17] and so they should be considered as the more valid of the calculations (assuming that the other force-time analysis methods that we have recommended in our methods section are used). 

## 5. Conclusions

The FT:CT ratio and RSI_mod_ (both methods) demonstrate similar between-day reliability (all good-excellent based on CV% results) and share an almost perfect positive relationship. This is because the constituent parts of each calculation are either the same (i.e., CT) or they too demonstrate an almost perfect relationship. As such, either the FT:CT ratio or RSI_mod_ can be used to compare within- and between-athlete CMJ performances, however there is little reason to include both variables. Researchers and practitioners should be mindful, however, that accurate FT values depend on athletes not tucking their legs during the flight phase of the CMJ. This problem will affect the FT:CT ratio and RSI_mod_^FT^ values, however it will not affect RSI_mod_^TOV^ values. Therefore, it is recommended that, where possible, researchers and practitioners use RSI_mod_^TOV^ for monitoring changes in athletes’ CMJ force-time characteristics between testing occasions, however future empirical studies that are conducted longitudinally within a sports setting are required to validate this recommendation.
